# Monthly Summary

**Published:** 1867-07

**Authors:** 


### MONTHLY SUMMARY.

*Diseased Meat.*—Dr. Letheby has recently published an interesting paper on this subject from which we glean the leading facts.

The Doctor superintends the inspection of butcher's meat for the city of London, and some idea of the labours performed by the inspectors may be gathered from the fact that, during the six years in which the examination of meats has been conducted, nearly six hundred tons have been condemned and driven out of the market. The inspectors have been instructed to seize

1. The carcases and joints of all animals which have died from accident or disease.

2. The meat of animals showing signs of acute inflammatory disease, and of recent plastic exudation, for instance, of all exhibiting traces of pleuro-pneumonia.

3. The meat of all animals slaughtered during parturition.
4. All meat infected with parasitic disease.
5. The flesh of animals tainted with physic.
6. The meat of animals wasted by lingering disease, being serous, pale, wet, and flabby.
7. All meat in a high state of putrefaction.

Dr. Letheby gives several distinguishing marks of good and bad meat.

Thus, good meat is neither of a pale pinkish color, nor of a dark purple tint. The former indicates disease, the latter natural death, or acute fever. Good meat is marbled by small veins of fat which is hard and suety, and never wet; while that of diseased meat is soft and watery, often like jelly or sodden parchment. Healthy meat feels firm and somewhat elastic and scarcely moistens the fingers, while diseased meat is soft and wet, often so much so that serum runs from it. Good meat has little odour and that not disagreeable, while diseased meat smells faint and cadaverous, and often emits the odour of medicine, especially when fresh cut, or minced and treated with hot water.

Good meat shrinks little in cooking, while bad meat shrivels or boils to pieces. These differences are owing to an undue proportion of serum in the meat, and to excess of gelatinous or intercellular tissue, fat and true muscular fibre being more or less deficient. The average loss of weight of sound beef, on drying, is 73.3 per cent. and of mutton 71.5 per cent. while diseased beef loses 76.1 per cent. and bad mutton 78.2 per cent. The juice of good meat is slightly acid and abounds in potash, while that of bad meat is alkaline and soda predominates in it.

The relations of bad meat to disease in those who eat it are somewhat obscure. We know indeed that *trichina* can communicate itself to man, and that *cysticercus* in pork or mutton becomes tape-worm in human intestines. It has been asserted, however, on good authority, that diseased meat may be eaten with impunity. Dr. Letheby, however, traced an endemic of sickness, vomiting and great prostration of strength to some sausages made from a cow slaughtered while laboring under pleuro-pneumonia. He quotes the African traveller, Dr. Livingstone, to the effect that the meat of animals slaughtered while suffering from this disease, produces malignant carbuncle in those who eat it. He corroborates this statement by citing statistics to show that after the importation of pleuro-pneumonia in cattle from Holland, deaths from carbuncle increased more than six-fold, and the mortality from phlegmon multiplied thirty-fold. This ill effect of this kind of diseased beef cannot be destroyed or mitigated by boiling or roasting.

---

*Reproduction of Bone.*—Much has of late years been said of the power of periosteum to restore great waste of bone. We find in a recent number of the *Boston Medical and Surgical Journal* a case reported by Dr. Henry J. Bigelow which is so instructive that we make space for a brief abstract.

A light-haired, unhealthy looking man, of a scrofulous family, injured his elbow which swelled and gave him great pain. Such swelling and pain always accompanied all subsequent injuries of the same joint, for five years. Towards the close of that period, fistulous openings made their appearance, communicating from one side of the limb to the other, and discharging a thin sanious liquid. The incision revealed in the humerus a cavity, the size of an almond, lined with caries. Three months afterwards, the formation of abcesses having continued, the joint was opened. The ends of all three bones were much diseased, and the head of the radius, together with about an inch of the ulna and the same amount of the humerus, was excised. The periosteum, being firmly attached to the coral-like surface of the bones, was torn out from these inequalities, and the wound was closed, the periosteum being allowed to remain within. The constitutional irritation still continuing, five months after the excision, the arm was amputated. About a year following the amputation, the man died of pulmonary consumption.

Upon dissection of the amputated arm, it was found that both the condyles of the humerus, as well as the process for the attachment of the flexors and extensors had been reproduced by the periosteum.

---

*The Convertibility of Electricity and Heat* is illustrated by joining a bar of antimony and another of bismuth, end to end, and passing a current of electricity through them, first from the one end and then from the other. The current passing from the antimony to the bismuth will be found, by proper tests, to part with a portion of its electrical intensity at the junction, and to develope increased heat. That passing from the bismuth, on the contrary, will manifest increased electrical tension, evidently at the expense of the pre-existing heat, for the bar at that point will be colder than before the current passed. The same principle has been applied by General Morin, so as to produce a self-registering electrical thermometer. A thermo-electric battery—developing electricity by the application of heat—is arranged with one extremity of the pile in a medium of uniform and low temperature (ice) and the other in the medium the temperature of which is to be measured. A needle is magnetized by the thermo-electric current produced by this temperature, and its consequent deflection from a certain natural position is registered by punctures made by a dial of paper which is caused by clockwork to complete a revolution in twenty-four hours, and also to rise to meet the puncturing point at equal intervals, hours, half hours, etc., as may be desired. The punctures made at the several hours will indicate by their variation from a circle, the changes of temperature throughout the day.

---

*Charcoal.*—The interesting mystery of the power of charcoal to absorb, condense and change gases to vapors, engages continued investigation. Among the latest observations reported, the remarkable chemical activity in charcoal saturated with oxygen is displayed in the conversion of moist sulphurous acid and sulphuretted hydrogen to sulphuric acid; common alchohol to acetic acid, and amylic alcohol to valerianic acid; indicating a power of oxidation extending to a very wide range of effects, but to which ammonia showed an exception. The condition in which oxygen exists so largely and actively in charcoal is yet a mystery.

---

*Formation of Cells in Animal Bodies.*—Mr. E. Montgomery has communicated to the Royal Society a paper on this subject, in which he removes even cell-growth back among the processes actuated by the physical and chemical forces alone.

He calls attention first to the fact that cells of low organization, chiefly those of cancerous tumours, upon the addition of water, expanded far beyond their ordinary limits and at last were lost amid the surrounding medium. He thinks this phenomenon explicable only on the assumption that the so-called cells are really uniformly viscid globules which absorb water till they are finally liquefied. The nuclei did not always show this change.

In embryonic tissues and in various tumours, single nuclei were seen, each surrounded by a shred of granular matter. On the addition of water, sometimes a bulge would take place on the edge of the granular mass, increasing to a globule and finally floating off; at others, the whole granular mass would turn into a globule inclosing the nucleus in its centre, and assuming the form of a typical cell. Sometimes again, several nuclei were connected with a granular mass, which, developing into a globule, enclosed them all, resembling in final developement, the "mother cell." The cell, therefore he reckons as really a viscid globule.

In searching for a substance which would furnish such results, he finally hit upon myeline. To this, dry and amorphous, water was added and from all the free margins, slender tubes, sometimes closely resembling nerve-tubes, shot forth. When white of egg was added, globules weie formed. With serum, the globules exhibit lively molecular motions.

The author thinks he has definitely ruled out the vital force. We cannot echo his shouts of triumph. It remains to be proved first that living cells are always viscid globules; secondly that myeline will always generate nerve-tubes and globules by its own inherent forces, and thirdly that myeline is the foundation of all living tissue. When this is done, Mr. Montgomery will have tc account for the reproduction of similar tissues from similar living membranes e. g. bone from periosteum, &c. We think our author's jubilation a little premature.

---

*The Air Treatment.*—M. Boisson has introduced a method of treating superficial wounds by a jet of air from the common bellows, immediately forming a dried film over the exposed flesh, beneath which healing is greatly facilitated and other obvious advantages secured. Burns which have removed the skin may be treated advantageously in this way.

---

*Physiology of Respiration.*—Pettenkofer has developed some very remarkable facts in connection with this important function.

He has found that there is a decimal variation in it, analogous to that which obtains in plants. The last named organism evolve oxygen during the day and carbonic acid at night. Animals exhibit on this respect their customary antithesis to vegetables. They evolve carbonic acid in large quantities during the day, wholly irrespective of muscular activity, while at night they lay up a store of oxygen for the requirements of the following day. Experiments on marmots show that these animals actually increase is weight during their winter sleep, and in the case of a man who occupied Pettenkofer's apparatus for two nights, it was shown that during the hours of darkness he absorbed 200 grammes more oxygen than he exhaled carbonic acid. Hence it is apparent that there is no direct oxidation of tissue or food into the ultimate products of combustion, water and carbonic acid. Indeed it is now known that this change does not take place immediately even in ordinary combustion. Intermediate products are formed in both cases, these being of course most numerous in the slower oxidation in the animal body.

There appears to be a direct relation between the injection of albumen and the absorption of oxygen. The more albumen is taken in with the food, the more oxygen is absorbed at night, and the less albumen is consumed, the less capacity has the body for taking of oxygen during sleep. In diseases of defective assimilation the storage of oxygen is heavily diminished.

The blood corpuscles, to use a simile of Pettenkofer's are a fleet laden with oxygen and carried to the most distant organs, when part of it is used up for the work actually going on and part is stored for future use. Carbonic acid constitutes the return freight of these minute vessels, which, miscroscopic as they are, nevertheless carry forward and backwards a load of four and a half pounds of each of these cargoes. By day they export carbonic acid; by night they import oxygen and carry it to every part of the system, so that it may be ready throughout the organism to supply the combustion of the following day.

---

*Influence of Alchohol on temperature of the Body.*—Drs. Ringer and Ricker state, as the result of a series of experiments on this question, that alcohol in poisonous doses decidedly lowers the temperature of persons in health. In ordinary doses it slightly depressed the temperature in eight out of eleven cases. In febrile conditions its effect on the temperature of the body is both trifling and temporary.

---

*Ether Spray in Strangulated Hernia.*—Dr. John Barclay, reports in the British Medical journal, a case of strangulated hernia, in which reduction was accomplished after the use of ether spray. The pain induced by the most gentle handling of the hernial tumor was so intense, that Dr. B. had to desist from taxis. Having brought with him Richardson's ether spray apparatus, thinking it might be useful in lieu of ice,—it was determined to invert the patient, apply the ether spray short of freezing the skin, then to attempt the reduction, and, if failure was the result, to operate by the knife.

---

